# Biopolymer-Based Hybrids as Effective Admixtures for Cement Composites

**DOI:** 10.3390/polym12051180

**Published:** 2020-05-21

**Authors:** Agnieszka Ślosarczyk, Izabela Klapiszewska, Patryk Jędrzejczak, Łukasz Klapiszewski, Teofil Jesionowski

**Affiliations:** 1 Institute of Building Engineering, Faculty of Civil and Transport Engineering, Poznan University of Technology, Piotrowo 3, PL-60965 Poznan, Poland; izabela.klapiszewska@put.poznan.pl; 2 Institute of Chemical Technology and Engineering, Faculty of Chemical Technology, Poznan University of Technology, Berdychowo 4, PL-60965 Poznan, Poland; patryk.j.jedrzejczak@student.put.poznan.pl (P.J.); teofil.jesionowski@put.poznan.pl (T.J.)

**Keywords:** cement composites, admixtures, biopolymers, lignin-based hybrid materials, dispersive and mechanical properties

## Abstract

In the framework of this publication, silica-lignin hybrid materials were designed, obtained, characterized and then used as admixtures for cement composites. High-energy mechanical grinding of individual components was used to produce the systems that allowed ensuring adequate homogeneity of the final products. As a result of the analysis of Fourier transform infrared spectroscopy, it has been confirmed that weak physical interactions occur between the components. This allowed classifying the resulting systems as I class hybrid materials. In addition, the efficiency of obtaining final products was also inferred on the basis of obtained porous structure parameters and colorimetric data. The achieved bio-admixture with different weight ratios of silica and lignin was added to cement pastes in the amount ranging from 0.5 to 1 wt.%. The study showed that increasing the ratio of lignin in the admixture from 0.15 to 1 wt.% had a positive effect on the rheological properties of the pastes, while the mechanical properties of the composite were deteriorated. In turn, a higher amount of silica in the admixture acted in reverse. The most favorable results were obtained for a silica-lignin bio-admixture with a weight ratio of components equal to 5:1 wt./wt. A significant increase in compressive strength was gained at satisfactory plasticity of the paste.

## 1. Introduction

Biopolymers were already used as admixtures in construction materials in ancient times. The first reports regarding the use of vegetable oils in lime mortars appeared in the works of Vitruvius. The Romans also used other bio-admixtures, such as dried blood to aerate building materials or proteins as gypsum binding regulators. In turn, the Chinese used bio-admixtures such as proteins, egg whites, fish oils or blood to modify the mortar used during the construction of the Great Wall [1,2]. The durability of the buildings founded at that time, some of which have survived to this day, indicates the validity of the use of biopolymers in present-day technology of construction materials. This is also important because of the increased search for biodegradable materials from natural sources as an alternative to polymers traditionally synthesized from petroleum products. Although the production of bio-admixtures is still relatively low compared to petroleum-based ones, the use of bio-admixtures is expected to soon increase significantly. This is related to the global trend of sustainable development and dissipation of natural resources. It is also of ecological importance, related to the need to look for biodegradable materials that will not be a problem or an ecological threat, which is currently occurring due to the large amount of plastic-based materials being released into the environment [3,4].

Taking the construction industry into account, currently, biopolymers are most often used to modify the properties of concrete and dry-mix mortars. Contemporary concrete does not exist without polymer modifications, including polymer-based chemical admixtures, which influence many of the characteristics of concrete, such as rheology, strength and durability. The global annual concrete production exceeds 10,000 million tones, making the share of chemical admixtures, including biopolymers, very high. It is predicted that the demand for concrete and bio-admixtures will extensively increase in the near future due to the intensive growth of the construction industry in many developing countries [5,6]. 

Bio-admixtures may be obtained directly from natural sources or via modification of natural products (e.g., cellulose, starch, chitin, chitosan or alginates) [7,8,9,10]. They may be also obtained in the fermentation processes carried out by certain bio-organisms (e.g., polyhydroxyalkanoates) and by biosynthesis (e.g., polylactide, bio-polyethylene, polytrimethylene terephthalate) [11,12]. Nevertheless, the most common and most widely used biopolymer in construction is lignin and its derivatives, such as lignosulfonate, mainly due to low costs, accessibility and the possibility of producing large quantities at an industrial scale [13,14]. Lignosulfonates have been used as plasticizing admixtures for mortars and concrete in concrete technology since the 1960s. They allow reducing water content by approx. 15%, which, on the one hand, increases the workability of the concrete mix while maintaining constant water to cement ratio, and on the other hand, allows to obtain higher compressive strength of the concrete while reducing the amount of water [14,15]. In modern concrete technology, lignosulfonates are increasingly being replaced by cellulose ethers such as carboxymethyl cellulose and hydroxyethyl cellulose, which allow for a much greater reduction in water, up to 40%, and significant strength gains of concrete structures [16,17]. However, such a solution is more expensive due to the higher production costs of cellulose ethers compared to lignosulfonates, which in turn are a by-product of the cellulosic industry [18]. 

In addition to improving flowability and workability, lignin and its derivatives can have dispersive functions and can be applied as carriers for a variety of active ingredients [19]. Such materials should be characterized by good solubility and release characteristics. Kraft lignin, for example, is well soluble in aqueous alkaline solutions and certain mixtures of organic solvents such as aqueous solutions of acetone or tetrahydrofuran, while lignosulfonates are soluble in aqueous solutions over a wide pH range [19]. Bio-refined lignin behaves differently depending on the purification process and the variability of feedstock materials. The high degree of purification of the raw material also determines the possibility of using lignin and its derivatives in medicine, while products with a lower degree of purification can be used in technical applications [15,19]. The introduction of active substances onto/into lignin and their derivatives usually occurs through entrapment, encapsulation and adsorption processes. These processes are most often used in medicine and biotechnology, e.g., to introduce certain drugs, enzymes or pesticides [20,21,22,23]. In technical applications, such solutions are still very limited. 

It is well known that the main role of silica is to increase the compressive strength of cement composite. The increase in strength is related to the reaction of the silica with the calcium hydroxide produced during the hydration of the silicate phases of cement clinker. This results in the creation of an additional calcium silicate hydrate (CSH) phase which seals the cement paste. Much better results are obtained for silica with nanometric dimensions, which unfortunately tends to aggregate and agglomerate. This results in the lack of good dispersion of the nanoadditive in the cement matrix and its lower efficiency. Therefore, in recent years, particular attention has been paid to the proper dispersion of silica, especially nanosilica, in cement-based composites. One way is to disperse the silica in water by means of ultrasound or to use liquefying and plasticizing admixtures [24,25,26,27,28]. 

Due to the good dispersing properties of kraft lignin in alkaline solutions and the very good plasticizing properties of lignosulfonates, as well as their wide use in the construction sector, an admixture based on a biopolymer (kraft lignin) and silica with different weight ratios was synthesized in this study. The main aim of the research was to check how the composition of the bio-admixture (ratio of silica to lignin) will affect the physical and mechanical properties of the cement composite. The main role of silica is strengthening of the cement paste, but very often silica aggregates and agglomerates, hence some of its properties is lost. The combination of silica and lignin should counteract this phenomenon. For this purpose, a wide range of dispersive and morphological properties of the bio-admixture itself was carried out and its effect on the rheological and mechanical properties of the cement paste was assessed.

## 2. Materials and Methods 

### 2.1. Materials

Syloid 244 was used in the present study. This is fine particle size, synthetic amorphous silica produced by W.R. Grace and Company (Columbia, MD, USA). In addition, kraft lignin (Sigma-Aldrich, Steinheim am Albuch, Germany) with an average molecular weight equal to Mw ~10,000 was used. The cement that served as the basis of the study was Portland CEM I 42.5R, Górażdże Cement SA, Górażdże, Poland, which included Portland clinker (95%) as the main component and a setting time regulator (up to 5%).

### 2.2. Preparation of Silica-Lignin Hybrids

In order to obtain first class hybrid materials, a method of mechanical grinding of components was used. We have already employed this method in our previous publications [29,30,31,32]. This method involves mechanical grinding of precursors, with simultaneous mixing using (i) RM100 mortar grinder (Retsch GmbH, Haan, Germany) and (ii) Pulverisette 6 Classic Line ball mill (Fritsch GmbH, Idar-Oberstein, Germany). The combined application allows obtaining final products characterized by adequate homogeneity. As part of this publication, silica-lignin hybrid systems with the following weight ratio of inorganic to organic parts were prepared: 5:1, 2:1, 1:1, 1:2 and 1:5.

### 2.3. Physicochemical and Dispersive-Microstructural Characteristics of Inorganic-Organic Hybrids

The Mastersizer 2000 analyzer (Malvern Instruments Ltd., Malvern, UK) was exploited to determine the particle size and dispersion data. The apparatus allows determining the particle size in the range of 0.2–2000 µm, using the laser diffraction method. 

Surface morphology, as well as the shape and size of individual particles were examined using scanning electron microscopy (SEM). The EVO40 microscope from Zeiss AG, Jena, Germany was applied for the research. Prior to taking a picture of the surface of the material, the sample was covered with gold using a Balzers PV205P apparatus (Oerlikon Bazers Coating SA, Biel, Switzerland).

In order to confirm the effectiveness of the preparation of hybrid materials, Fourier transform infrared spectroscopy (FTIR) was accessed. In the FTIR method, infrared radiation covers the range of 4000–400 cm^−1^. Compound spectra were obtained using a Vertex 70 spectrometer from Bruker Optik GmbH, Ettlingen, Germany. This camera is the first fully digital spectrometer that offers a wide spectral range (up to 25,000 cm^−1^) and a resolution better than 0.5 cm^−1^.

A Specbos 4000 colorimeter (JETI Technische Instrumente GmbH, Jena, Germany) was utilized to measure the color of the materials. This apparatus determines the basic colors in the 0°/45° geometry surface and differentiates the tested powder materials in terms of even slight discrepancies occurring in the shades of colors. As part of the study, the CIE L*a*b* color system was employed.

### 2.4. Preparation of Cement Pastes

In order to obtain cement paste, the following components were weighted and mixed: 150 g of Portland cement CEM I 42.5 R, 75 cm^3^ of distilled water and 0.75 g of admixture (in the case of samples containing 0.5 wt.% admixture) or 1.5 g of admixture (in for samples containing 1 wt.% admixture).

**Figure 1:**
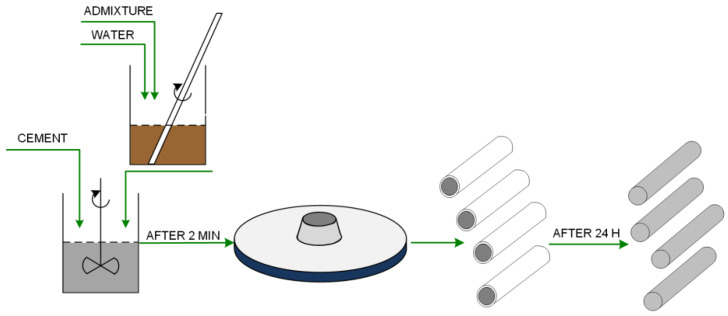
The scheme for obtaining cement paste samples.

The cement was placed directly in the stirrer cuvette, while the appropriate amount of admixture was first dissolved in distilled water and then poured into the cuvette. All prepared ingredients were subjected to vacuum mixing in an automatic mixer from Renfert, Hilzingen, Germany, for 2 min at 450 rpm. The prepared paste was tested on a shaking table and then placed in standardized containers with a release agent applied to obtain the rollers necessary for the strength tests. After 24 h, the samples were demoulded and allowed to cure in water for 28 days. A schematic diagram of the preparation of cement paste samples is shown in Figure 1.

### 2.5. Cement Pastes Tests

#### 2.5.1. Mixture Consistency

The test consists of determining the flow diameter of the paste sample on a shaking table. The test paste was placed in a mold in layers, thickening each layer with impacts of the beater. The mold was then lifted vertically and the sample was shaken by turning the crank 15 times. Immediately after shaking, two perpendicular diameters of the spilled leaven cake were measured. The diameter of the spilled cake is the measure of consistency.

#### 2.5.2. Compressive Strength Test

The cylinder sample strength tests were carried out using an Instron testing machine, Satec, Norwood, Massachusetts, USA. This is a static machine, designed for testing samples under compression, bending and stretching.

The test was carried out according to PN-EN 196-1: 2006 “Cement testing methods. Part 1: Determination of strength.” The test cylinders were characterized by standardized dimensions of 2 cm × 2 cm. The sample was placed between two compression plates that tightly clamped the central part of the sample under test. The load was evenly increased and recorded until the sample was crushed. The compressive strength test was carried out on samples after 28 days of seasoning.

#### 2.5.3. Microstructural Analysis

The surface morphology and structure of the solid sample were assessed using an EVO40 scanning electron microscope with a natural breakthrough after mechanical testing. As with powder samples, the surface of the sample was previously covered with gold.

## 3. Results and Discussion

### 3.1. Physicochemical and Dispersive-Microstructural Characteristics of Inorganic-Organic Hybrids

#### 3.1.1. Dispersive-Microstructural Properties

**Figure 2:**
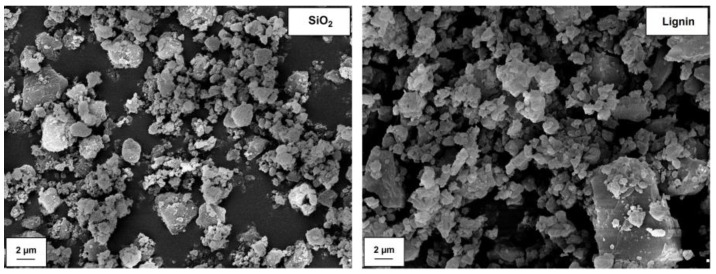
SEM microphotographs of pristine components (silica and lignin).

**Figure 3:**
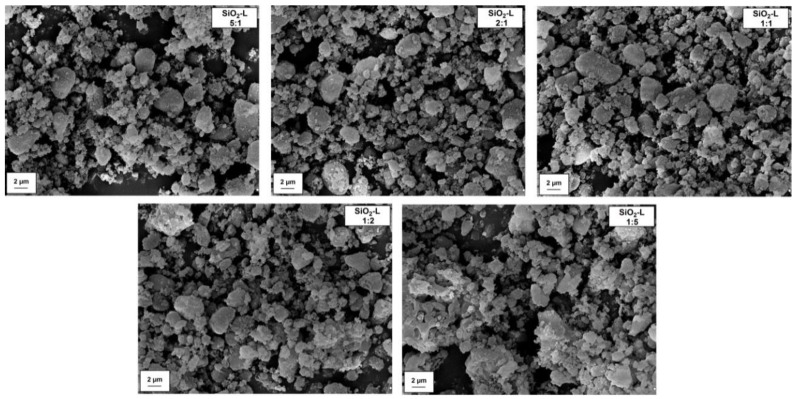
SEM microphotographs of silica-lignin hybrid materials with different weight ratios of the components used.

SEM images for pristine silica Syloid 244 and kraft lignin are compared in Figure 2. According to the manufacturer’s data, similarly to the observation of the microphotography, silica is characterized by particles in the nanometric range. However, they exhibit a significant tendency to form aggregates (<1 μm) and agglomerates (>1 μm). In turn, the lignin particles possess a larger size compared to silica, as evidenced by the presence of large particles, reaching the size of even 6–7 µm. Dispersion data obtained on the basis of measurements using the Mastersizer 2000 apparatus, indicating that the average particle size for a lignin sample is equal to 6.5 µm (see Table 1), are a confirmation of these conclusions. In turn, based on the same table, it can be concluded that silica possesses an average particle size of 1.9 µm. This clearly confirms that, despite the presence of particles below 100 nm in the SiO_2_ structure, their tendency to form larger clusters is significant. In addition, SEM images of obtained silica-lignin hybrid materials are presented in Figure 3. On the basis of the microphotography, it can be concluded that the products exhibit an increased tendency to form larger structures as the lignin content in the hybrid increases. This is particularly evident in the case of hybrid material SiO_2_-lignin 1:5 wt./wt. The conclusions are confirmed by dispersion data presented in Table 1. These data show that the average particle size for hybrid systems is in the range of 2.3–4.8 µm, and the highest value is achieved for the silica-lignin system with a component weight ratio of 1:5.

In our previous papers, we also presented the most important parameters of the thermal stability, as well as porous structure for selected silica-lignin hybrids and pure components [33,34,35]. The highest BET surface area was observed for pristine silica Syloid 244 (A_BET_ = 262 m^2^/g). In turn, pristine lignin possesses a very small BET surface area—1 m^2^/g. The biopolymer is also characterized by a total pore volume value of 0.01 cm^3^/g and an average pore size of 12.1 nm. Based on the data presented in [33,35], it can be concluded that the BET surface area values decrease as the ratio of lignin in hybrid systems increases. The situation is similar in the case of the total volume of pores. The converse is the case with the mean size of pores. The obtained values are consistent with the expectations and indirectly indicate the efficiency of hybrid materials.

#### 3.1.2. Fourier Transform Infrared Spectroscopy

The list of absorption maxima for individual vibrational assignment at the right wavenumber, obtained on the basis of Fourier transform infrared spectroscopy, is presented in Table 2. The obtained data confirm the identification of the relevant components (silica and lignin), as well as the efficiency of their interconnection.

For pristine silica, a band with a maximum of 3450 cm*^−^*^1^ was recorded, derived from the stretching vibrations of O–H bonds that are exemplary for physically bound water. In addition, subsequent bands reveal typical vibrations characteristic for silica: Si–O group (ν_s_: 1200 cm*^−^*^1^; ν_as_: 1102 cm*^−^*^1^), Si–OH group (ν_as_: 992 cm*^−^*^1^, ν_s_: 865 cm*^−^*^1^) and Si–O group (δ: 512 cm*^−^*^1^), where ν_s_ and ν_as_ are symmetrical and asymmetrical stretching vibrations and δ–bending vibrations, respectively. The obtained absorption maxima data are consistent with the course of the silica spectrum, which were included in previous publications [33,36,37,38].

In turn, lignin possesses characteristic bands at the following maxima: 3410 cm*^−^*^1^ (band of stretching vibrations of O–H bonds); 2890 cm*^−^*^1^ (band of stretching vibrations of C–H bonds, among others –CH_3_, –CH_2_ and –OCH_3_), 1670 cm*^−^*^1^ (stretching vibrations of C=O bonds), 1596 cm*^−^*^1^, 1518 cm*^−^*^1^, 1480 cm*^−^*^1^ and 1445 cm*^−^*^1^ (stretching vibrations of C–C and C=C bonds of the aromatic ring), 1250 cm*^−^*^1^ and 1208 cm*^−^*^1^ (stretching vibrations of C–O(H) and C–O(Ar) bonds) and 1094 cm*^−^*^1^, 1015 cm*^−^*^1^ (stretching vibrations of C–O–C bonds). The presence of all these bands is characteristic for pristine lignin and has also been confirmed by us in other publications, in the form of an appropriate spectrum [33,36,39].

In order to confirm the effectiveness of the preparation of silica-lignin hybrid materials, the absorption maxima of individual bands were determined and listed in Table 2. On the basis of the obtained data, small maxima shifts of individual bands can be clearly seen, indicating the formation of physical interactions between components in the form of hydrogen bonds. This type of connection is characteristic for class I hybrid systems and is sufficient when applying such products as potential cement admixtures. Similar conclusions have already been established within the framework of properly designed hybrid systems such as MgO-lignin [29], ZnO-lignin [30] or Al_2_O_3_-lignin [32].

#### 3.1.3. Colorimetric Analysis

**Figure 4:**
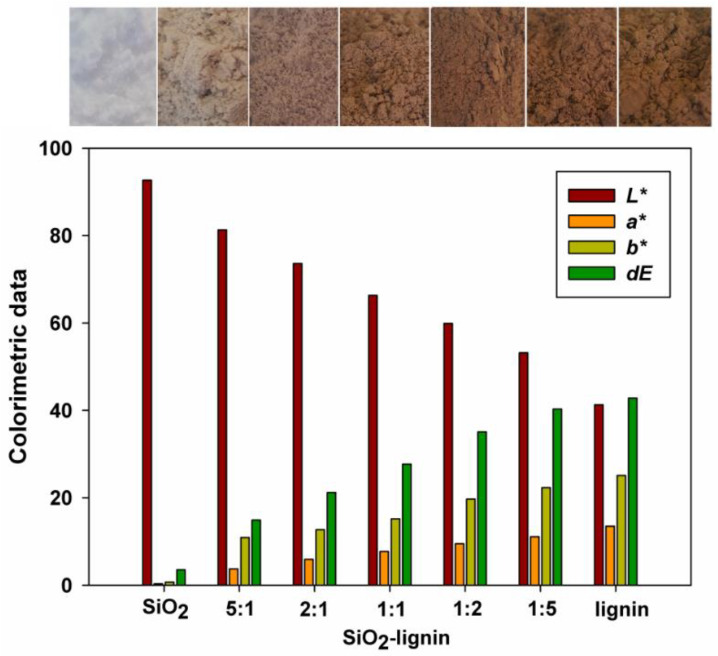
Colorimetric analysis of silica, lignin and silica-lignin hybrid materials with different weight ratios of the components used.

As part of the study, the color characteristics for the obtained hybrid materials and pristine components were also carried out (see Figure 4). Colorimetric analysis using the CIE L*a*b* color space system was performed to determine the lightness, the proportion of individual colors, saturation and hue. This type of analysis gains practical significance in application research in which the color of the material plays an important role. Pristine silica was used as a reference standard in the analysis. Syloid 244 used in the study is characterized by high lightness-L* parameter equal to 93.2. 

In turn, lignin is a dark brown solid, for which the parameter L* reaches the value 41.2. In the case of analyzing the values of the parameters a* and b*, which are responsible for the ratio of red and yellow in the lignin sample, values equal to 10.1 (parameter a*) and 25.9 (parameter b*) were observed. The dE variable is also an important parameter in the colorimetric analysis, as it determines the total color change, which is equal to 44.3 for used biopolymer.

Hybrid materials obtained on the basis of Syloid 244 silica are characterized by a progressive decrease in lightness (L* parameter) as the biopolymer content increases in the tested samples (see Figure 4). In case of the SiO_2_-lignin hybrid system (5:1 wt./wt.), the value of the parameter L* = 81.7 was noted, while the increase in the ratio of lignin in the hybrid (SiO_2_-lignin 1:5 wt./wt.) resulted in lowering the value of the L* parameter to 54.3. 

Based on the analysis of colorimetric data, a progressive increase in the value of the parameter determining the total color change (dE) was also observed. The highest value of this parameter was achieved for the SiO_2_-lignin 1:5 wt./wt. product and it was equal to 40.3. Analysis of the attached digital photos (see Figure 4) also allows us to observe the differences in color between individual systems.

Colorimetric analysis allowed to obtain satisfactory results for all types of hybrid materials. Thus, the effectiveness of the proposed research methodology has been indirectly confirmed.

### 3.2. Cement Pastes Properties

#### 3.2.1. Slump Test Results

In order to determine the consistency of the cement paste, the slump test was performed on a shaking table.

**Figure 5:**
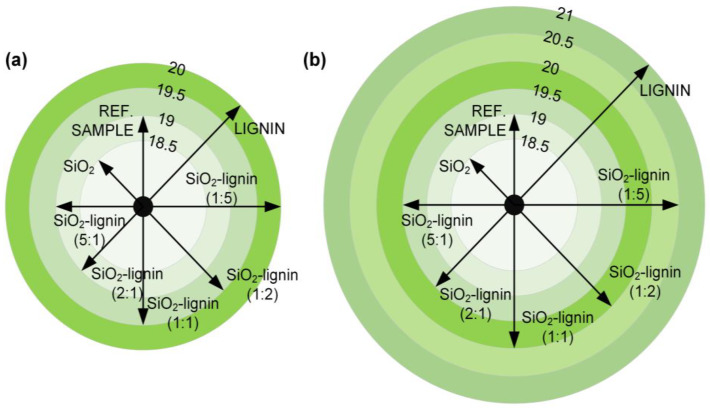
Slump test results for the samples containing 0.5 wt.% (**a**) and 1.0 wt.% (**b**) admixture.

**Figure 6:**
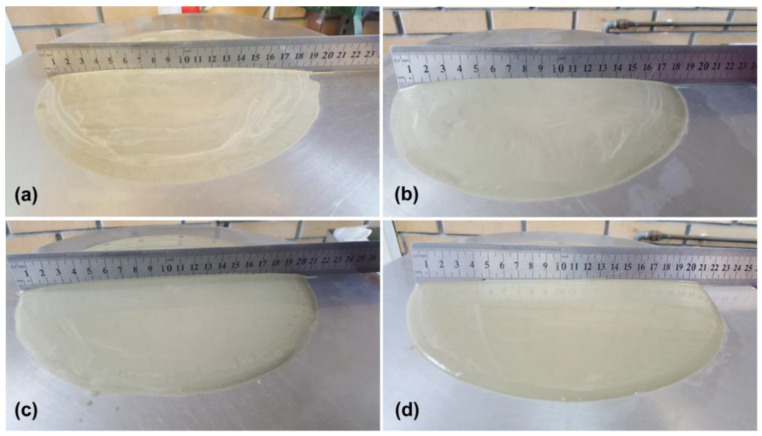
Digital photos of the slump for the reference sample (**a**) and the mixture containing 1 wt.% admixture of silica (**b**), silica-lignin hybrid material 1:5 wt./wt. (**c**) and lignin (**d**).

The size of propagation for the reference sample, without any admixture, is equal to 19 cm. Results for pastes containing 0.5 wt.% of admixtures are presented in Figure 5a, the samples containing 1 wt.% of the admixture are shown in Figure 5b, while digital images of carried outflows are shown in Figure 6. 

Accordingly, slump diameter for SiO_2_ admixture at 0.5 and 1 wt.% is equal to 18.5 cm, while for lignin it is equal to 20 and 21 cm, respectively. In the case of samples containing admixtures of hybrid materials, a beneficial effect of lignin on the increase in paste plasticity was observed. Therefore, the SiO_2_-lignin hybrid system–1:5 wt./wt. is characterized by the largest flow diameter, i.e., for samples with 0.5 wt.% admixture it is 20 cm, and for 1 wt.%–20.5 cm.

The authors of publications [40,41,42,43] also observed an increase in the plasticity of mixtures with the addition of lignin or its derivatives. Kalliola et al. [40] reported a beneficial effect of the admixture of oxidized lignin on the flow rate for pastes, mortars and concretes. Moreover, in their tests, it was confirmed that this admixture had no negative impact on the compressive strength of the mature concrete and the aeration of the mix. In their study, Li et al. [41] obtained lignin from pinewood, and then subjected it to a series of solvent modifications to obtain a lignin-based water reducer. The authors showed the influence of the sulfonation degree of the obtained lignin on the workability of pastes, including their plasticity. Arel [42] used lignosulfonate, a derivative of lignin, in his research, and prepared two series of samples with an admixture of 0.4 and 0.8 wt.% for two types of cement. In his research, he confirmed that the setting time, compressive strength and water reduction values increased together with the increase in lignosulfonate in the admixture. In their work, Klapiszewski et al. [43] showed that lignin had a more favorable effect than lignosulfonate on the mechanical properties of cement mortars. Increased plasticity was noted for both lignin and lignosulfonate samples.

#### 3.2.2. Compressive Strength Properties with the Silica, Lignin and Silica-Lignin Hybrid Materials 

**Figure 7:**
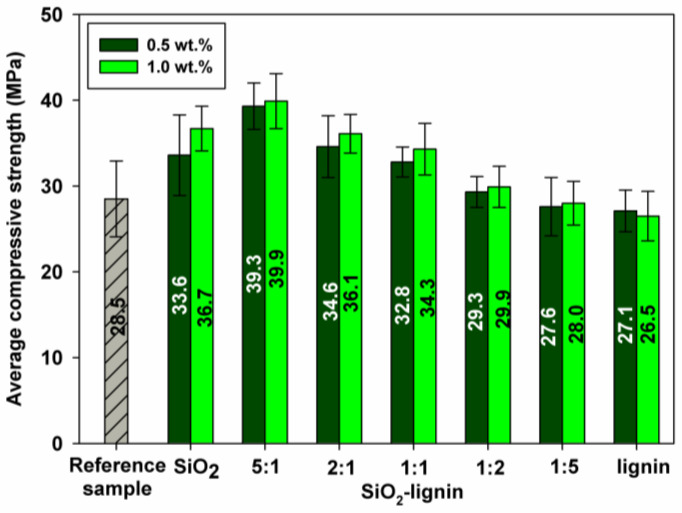
Compressive strength analysis of cement pastes with the silica, lignin and silica-lignin hybrid materials with different weight ratios of the components used.

**Figure 8:**
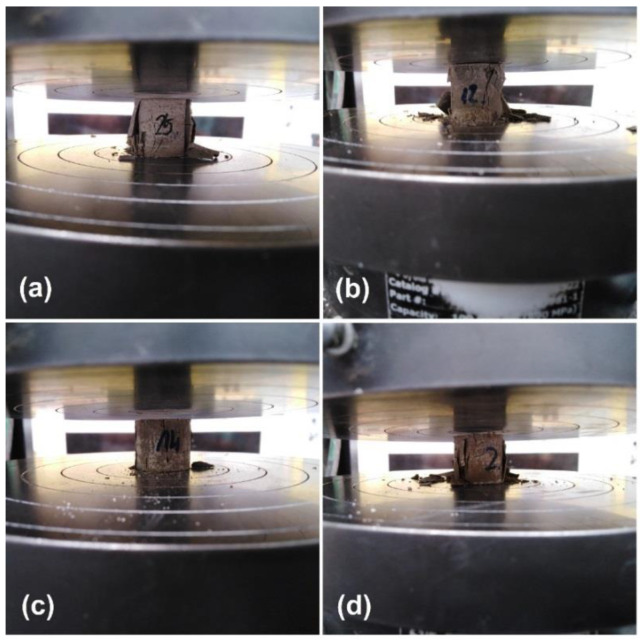
Digital photos of the samples after mechanical test for the reference sample (**a**) and the mixture containing 1 wt.% admixture of SiO_2_ (**b**), SiO_2_-lignin hybrid 1:5 wt./wt. (**c**) and lignin (**d**).

A comparison of the compressive strength results for pure cement paste and silica, lignin and silica-lignin hybrids at different weight ratios is presented in Figure 7. The admixtures were added to the cement paste in the amount of 0.5 and 1 wt.% in relation to the amount of cement. The lowest compressive strength was obtained for the sample with the addition of lignin alone. The results were slightly lower than the reference values obtained for pure paste without admixtures. With the increase in the amount of lignin in the composite, the value of compressive strength decreased, which is probably related to a greater aeration of the cement paste. Similar relationships have been reported by other researchers for lignosulfonates, hence the maximum amount of admixtures in cement composites is usually up to 0.5 wt.% in relation to cement [2,43,44]. As the bio-admixture in the system was replaced with silica, an increase in compressive strength was observed for both 0.5 and 1 wt.% samples. A noticeable increase in strength was observed for the silica-lignin system 1:1 wt./wt., while the highest compressive strength values were noticed for cement paste modified with the SiO_2_-lignin system (5:1 wt./wt.). Comparing the results for cement paste with pristine silica and lignin, it can be observed that the combination of both components into one bio-based admixture has a positive effect on both rheological and mechanical properties of cement paste, despite the significantly lower BET surface area of the hybrid system [33,35]. The combination of both materials has a very positive effect—an increase in the strength of the composite with less silica addition by almost 40%, compared to the reference sample, and on average by 11%–20%, compared to the lignin-free sample with silica. This confirms the dispersive properties of lignin and the beneficial effect on the physical and mechanical properties of the cement paste. In the case of the silica-lignin system, a better distribution of silica particles in the composite was obtained, which was confirmed by the plasticity test of cement composites presented in Figure 5. Herein, the spreads for the silica-lignin system (5:1 wt./wt.) were higher than those obtained for the reference sample and with pristine silica. Selected digital photos for samples after mechanical tests are shown in Figure 8.

#### 3.2.3. SEM Analysis of Cement Composite with the Silica, Lignin and Silica-Lignin Hybrid Materials 

**Figure 9:**
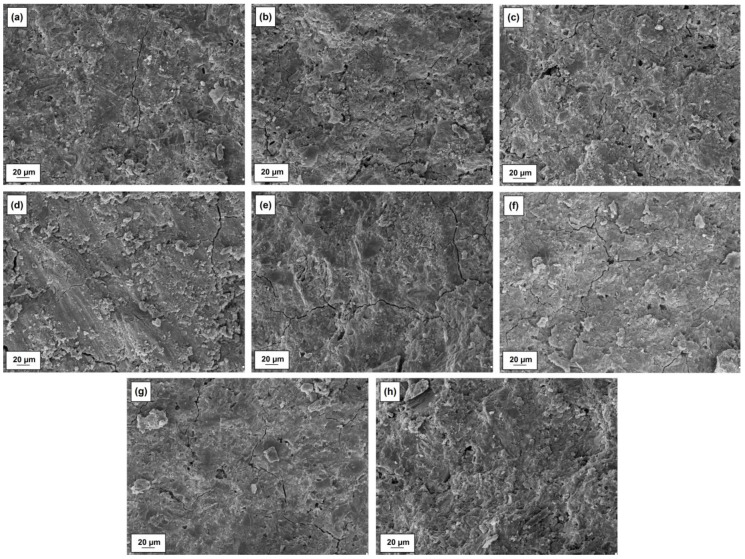
SEM images of pure cement mixture (**a**) and cement mixture with the addition of silica (**b**), lignin (**c**) and silica-lignin hybrid materials with different weight ratios of the components used–5:1 wt./wt. (**d**), 2:1 wt./wt. (**e**), 1:1 wt./wt. (**f**), 1:2 wt./wt. (**g**) and 1:5 wt./wt. (**h**).

**Figure 10:**
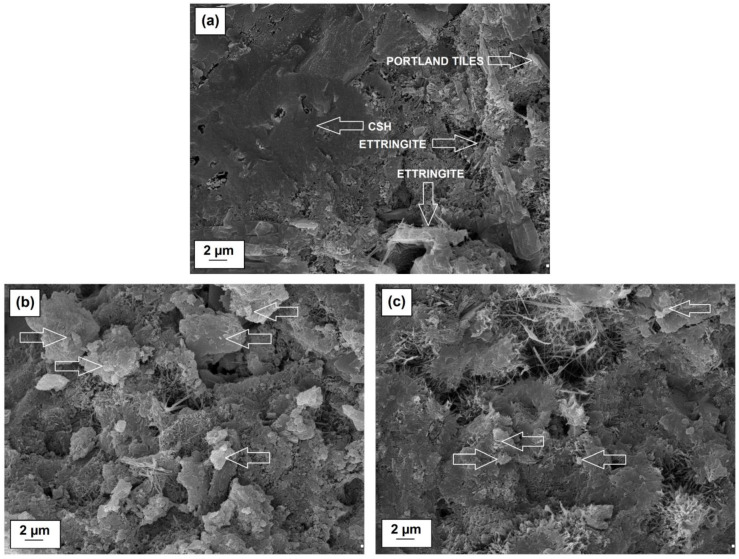
SEM images of pure cement mixture (**a**) and cement mixture with the addition of silica-lignin hybrid materials with weight ratios 5:1 wt./wt. (**b**) and 1:5 wt./wt. (**c**). (**b**,**c**)–hybrid particles are marked with arrows.

Microstructures of cement pastes with silica, lignin and silica-lignin systems as well as selected microstructures in magnification are shown in Figure 9 and Figure 10, respectively. 

The SEM image for a pure paste is typical for binders consisting of cement clinker. In case of the microstructure presented in Figure 10, a compact binder structure can be observed with characteristic products of hydration of the cement clinker: hydrated calcium silicates, the so-called CSH phase, Portlandite tiles and an elongated, bar-like shape ettringite [45,46]. When more than 0.25% by weight of lignin is added to cement paste (Figure 9c,f–h), small air bubbles are visible in the microstructure, evenly distributed throughout the binder. This results in a decrease in the compressive strength of the composite. With a higher ratio of silica in the hybrid system, from 1:1 wt./wt. composition to 5:1 wt./wt., and for paste with pristine silica itself, the amount of fine air bubbles is much lower (Figure 9b,d,e). The presence of higher amounts of silica, especially in a bio-admixture composition with a weight ratio 5:1 wt./wt., contributes to the microstructure’s density, which in turn results in significant compressive strength gains. Figure 10b,c show enlarged microstructures of cement pastes with silica-lignin bio-admixture (5:1 wt./wt. and 1:5 wt./wt.), which are marked with arrows. For a cement paste with a higher amount of silica, the SEM image shows evenly distributed silica particles in the whole volume of the paste, at distances ranging from 1 to several micrometers. For a 1:5 wt./wt. silica-lignin hybrid, the distances between adjacent smaller particles of bio-admixture are much greater, which indicates a worse dispersion in the paste.

## 4. Conclusions

An effective method of obtaining silica-lignin hybrid materials using the method of mechanical grinding of components was proposed. Weak physical interactions in the form of hydrogen bonds occurred between the components. This qualifies the resulting materials as a first class hybrid. In addition, based on dispersive-microstructural analysis, it was concluded that the silica-lignin hybrid materials possess primary particles in their structure that tend to aggregate and agglomerate. This process is more intense with the higher ratio of the biopolymer in the hybrid. In addition, determined properties of surface chemistry and values of porous structure parameters, as well as colorimetric data indirectly confirm the validity and effectiveness of the proposed method of obtaining hybrid systems.

It was established that the admixture based on the biopolymer and silica has a positive effect on rheological and strength properties of the cement paste. A significant improvement of compressive strength, by nearly 40% compared to the reference paste and 10%–20% compared to the paste with silica alone, was obtained for a silica-lignin bio-admixture at a 5:1 wt./wt. ratio. The combination of lignin and silica enabled good dispersion of silica in the cement paste which resulted in an improvement of the mechanical properties of the composite at a lower silica-lignin ratio. The paste with the admixture of this composition was also characterized by good microstructure density and low pore content. The admixtures with lignin content above 0.25 wt.% aerated the cement paste much more, which resulted in a decrease in compressive strength. In this case, the decrease in strength was also related to the lower ratio of silica in the admixture.

Summing up the research, it can be concluded that the proper design of the silica-lignin admixture composition leads to a product with specific properties-improved mechanical properties due to the presence of silica in the admixture and dispersing properties due to the presence of lignin. The main function of lignin is the proper dispersion of silica in cement paste, which exhibits a tendency towards aggregation and agglomeration as suggested by the particle size distribution. Therefore, particles of nanometric in size present in silica do not become active. The break-up of silica agglomerates by combining them with lignin has contributed to the positive results in terms of dispersing silica and a significant increase in the compressive strength of the cement paste.

## Figures and Tables

**Table 1:** Particle diameter of silica, kraft lignin and silica-lignin hybrids.

Sample	Particle Diameter from Mastersizer 2000 (μm)			
	d(0.1) *	d(0.5) **	d(0.9) ***	D[4.3] ****
Silica Syloid 244	0.9	1.5	2.6	1.9
SiO_2_-lignin (5:1 wt./wt.)	1.2	1.9	2.9	2.3
SiO_2_-lignin (2:1 wt./wt.)	1.2	2.1	3.2	2.5
SiO_2_-lignin (1:1 wt./wt.)	1.5	2.9	3.9	3.3
SiO_2_-lignin (1:2 wt./wt.)	1.8	3.6	4.6	4.1
SiO_2_-lignin (1:5 wt./wt.)	1.9	4.1	5.4	4.8
Kraft lignin	2.0	5.2	8.4	6.5

* d(0.1)–10% of the volume distribution is below this value diameter; ** d(0.5)–50% of the volume distribution is below this value diameter; *** d(0.9)–90% of the volume distribution is below this value diameter; **** D[4.3]–average particle size in examined system.

**Table 2:** Vibrational frequency wavenumbers (cm^−1^) for silica, lignin and silica-lignin hybrid materials with different weight ratios of the components used.

Sample	Vibrational Assignment								
	ν O–H	ν C–H_x_	ν C=O	ν C–C C=C	ν C–O(H) C–O(Ar)	ν Si–O	ν C–O–C	ν Si–OH	δ Si–O
	Wavenumber (cm^−1^)								
Silica Syloid 244	3450	-	-	-	-	1200 (s) 1102 (as)	-	992 (as) 865 (s)	512
SiO_2_-lignin (5:1 wt./wt.)	3447	2875	1660	1589 1515 1474 1442	1235 1196	1204 (s) 1105 (as)	1083 1002	995 (as) 867 (s)	515
SiO_2_-lignin (2:1 wt./wt.)	3441	2880	1659	1592 1518 1478 1443	1239 1198	1205 (s) 1105 (as)	1084 1005	996 (as) 868 (s)	516
SiO_2_-lignin (1:1 wt./wt.)	3434	2885	1664	1598 1521 1482 1448	1242 1203	1208 (s) 1107 (as)	1087 1008	993 (as) 867 (s)	520
SiO_2_-lignin (1:2 wt./wt.)	3432	2886	1667	1602 1525 1483 1449	1243 1203	1209 (s) 1109 (as)	1090 1010	998 (as) 871 (s)	519
SiO_2_-lignin (1:5 wt./wt.)	3428	2888	1671	1598 1520 1481 1447	1247 1205	1209 (s) 1110 (as)	1091 1012	1003 (as) 876 (s)	523
Kraft lignin	3410	2890	1670	1596 1518 1480 1445	1250 1208	-	1094 1015	-	-
